# Direct comparisons between hypnosis and meditation: A mini-review

**DOI:** 10.3389/fpsyg.2022.958185

**Published:** 2022-07-15

**Authors:** Gabriele Penazzi, Nicola De Pisapia

**Affiliations:** Department of Psychology and Cognitive Science, University of Trento, Rovereto (TN), Italy

**Keywords:** mindfulness, attention, absorption, contemplation, metacognition, cognitive control, consciousness

## Abstract

Hypnosis and meditation share phenomenological and neurophysiological features, and their comparison is a topic of growing interest in the scientific literature. In this article, we review a classification of these two kinds of non-ordinary states of consciousness, and discuss the studies that directly compare them. Some findings seem to suggest that hypnosis and meditation are distinct phenomena, while others underline their similarities, but experiments that directly contrast them are still scarce and no consensus has been reached yet. While this comparison could give us fundamental insights into central issues concerning the role of attention, metacognition and executive control in the study of consciousness, it is clear that we are still at the early stages of this research.

## Introduction

Hypnotic and meditative states are characterized by a series of changes in subjective experiences from the normal waking state, but the literature is still unclear on similarities and differences (Raz and Lifshitz, 2016).

Common characteristics are a state of general wellbeing and relaxation, accompanied by deep concentration, and mental absorption (Lynn et al., 2012). The induction of both states is used for clinical purposes, particularly when dealing with psychological problems such as depression, anxiety and mental stress, or to relieve chronic pain (Zeidan and Grant, 2016).

On the other hand, the procedures for reaching these states have profoundly different historical bases, and in many cases the reported phenomenology presents substantial differences, thus raising a large number of questions about their mutual positioning (Markovic and Thompson, 2016). A key issue that appears central is the understanding of the role played in both states by metacognition, i.e., the ability to represent, monitor, and control ongoing cognitive processes (Lush et al., 2016). While several meditative and specific hypnotic practices seem to train conscious metacognitive skills (Drigas et al., 2022), several hypnotic techniques seem to act more on basic cognition at an unconscious level, inducing a decline in metacognitive abilities (Dienes et al., 2016).

Given these premises, highlighting similarities and differences between these two states seems to be a precondition in the scientific understanding of human consciousness, to disentangle conscious from unconscious processing, as well as to elucidate the role of metacognition. While important theoretical comparisons are present in the literature (e.g., Raz and Lifshitz, 2016), direct experimental comparisons between hypnosis and meditation are an exception. In this article, we briefly start with a definition of hypnotic and meditative states, then discuss the main comparative theories, and then list the few studies that have attempted direct experimental comparisons.

## Phenomenology of hypnosis and meditation

In this section, we describe the main phenomenological aspects that separately characterize hypnosis and meditation.

### Phenomenology of hypnosis

Hypnosis is defined as a state of consciousness consisting of focused attention, reduced peripheral awareness, and increased responsiveness to suggestion (Elkins et al., 2015). A typical hetero-induced hypnotic session begins with the hypnotist inducing a state of relaxation in the subject, up to a state of true hypnotic trance. In this state, experience and behavior of the hypnotized subject are modeled in accordance with the hypnotic suggestions. Finally, the session ends with a de-induction procedure (for example a countdown) uttered by the hypnotist (Egner and Raz, 2007).

Hypnotic suggestions can induce a wide range of effects, implemented in therapeutic and experimental contexts, ranging from analgesia and other forms of sensory hallucinations to significant modulations of attentional and executive control processes (Terhune et al., 2002; Drigas et al., 2021). There are three main phenomenological components of the hypnotic state: dissociation, absorption and suggestibility (Cardeña and Spiegel, 1991).

Dissociation is the splitting of mental processes from the main body of consciousness, with simultaneous alterations in the sense of self in acting and volition. Subjects deeply immersed in a hypnotic state, when asked to perform a task in response to hypnotic suggestions, perceive a state of alteration in the sense of voluntariness, as if the tasks were performed outside of their own intentionality (Sadler and Woody, 2010).

Absorption, on the other hand, consists of a focused attention that fully engages the mental resources of individuals (Tellegen and Atkinson, 1974). Attention control processes are central to absorption in hypnosis, and therefore a crucial role is hypothesized for executive functions and the frontal lobes (Parris, 2017).

Finally, suggestibility is the ability to model behavior and subjective experience in accordance with hypnotic suggestions. Suggestibility as a trait in the hypnotic context is referred to as hypnotizability, and therefore as the individual's generic ability to experience what is suggested during hypnosis. For this purpose, specific scales have been constructed that allow the comparison between subjects with differences in the hypnotizability trait (Acunzo and Terhune, 2021).

### Phenomenology of meditation

The term meditation refers to a wide variety of contemplative practices, ranging from focused meditation to breath control, visualization or mantra recitations (Matko et al., 2021). These practices engage meditators in repetitive, specific mental trainings aimed at cultivating desired psychological qualities and peculiar states of consciousness. We can divide these practices into three general categories, even though in some protocols they can be used together.

In the first category, specific meditative practices regulate and exercise the meditator's attention toward a specific target (e.g., bodily sensations related to breathing) (Lutz et al., 2008). This concentration is characterized by a perceived state of absorption, with a phenomenological total involvement in a specific experience (e.g., breathing) or task (e.g., visualizing mental images), as opposed to the experience of being continually distracted by extraneous thoughts or stimuli (Pekala, 1991). One special form of focused meditation is mantra recitation, with the repetition of a sound, word or phrase to calm and focus the mind, as in Transcendental Meditation (TM) (Álvarez-Pérez et al., 2022).

The second category includes mindfulness meditation and open monitoring practices, where the attention is opened to present thoughts, sensations, images that come to mind, with the purpose of observing them in a detached and non-judgmental way in the present moment (Raffone and Srinivasan, 2010). Here there is a form of absorption as well, but the mind is not focused on a specific target, instead it operates widely, and the practice is oriented toward the cultivation of meta-awareness (an aspect of metacognition), i.e., the awareness of everything that happens in our experience (Smallwood and Schooler, 2015; Mooneyham and Schooler, 2016).

A third category of meditation practices acts not on the level of individual constructs as in the previous groups, but on the whole self of the practitioner. In this family of practices, sometimes referred to as deconstructive (Dahl et al., 2015), the main activity is to introspectively explore the nature and dynamics of self-related mental processes. This practice unveils a transient or illusory nature of self-representations, which can lead to a progressive deconstruction of the ego's sense and to egolessness (Epstein, 1988).

## Theoretical and methodological comparisons between hypnosis and meditation

In this section, we describe the similar and dissimilar phenomenological aspects of the hypnotic and meditative states.

### Similarities between hypnosis and meditation

A first overlap between hypnosis and meditation concerns bodily relaxation. Both conditions are states of consciousness achieved by induction procedures that improve relaxation, with corresponding physiological effects. Both hypnosis and meditation show a reduction in sympathetic responses and an increase in parasympathetic tone, although these generalized results are not supported by all the field studies (Tung and Hsieh, 2019; Fernandez et al., 2021).

Secondly, in both states relaxation enhances the development of an effortlessly absorbed state of attention in the present moment. However, it is not yet clear whether the states of hypnotic and meditative absorptions are identical phenomena. In meditative practice, avoiding distractions requires training and effort in novices; in a hypnotic session, individuals are instead absorbed in the suggestions apparently without effort. Some authors underline the psychometric ambiguity of the absorption construct evaluated by the self-assessment questionnaires, suggesting the need to identify a finer level for the definition of different types of absorption (Terhune and Jamieson, 2021).

Lastly, functional magnetic resonance imaging (fMRI) studies that have examined meditation or hypnosis separately show that these states are associated with similar and partially overlapping activation changes in frontal, salience, and default-mode networks, known to be involved in attentional and executive functions (McGeown, 2016). However, these similarities are very difficult to generalize due to the different forms of techniques and tasks included in these two families of practices.

### Differences between hypnosis and meditation

A first important difference concerns the methodology: in hypnosis, it is central the hypnotherapist's ability to induce the hypnotic state, whereas in meditation the emphasis is on the autonomous mental practice of the meditator, although both states can be led by real people or audio guides (Häuser et al., 2016; McClintock et al., 2019).

Another difference is that hypnosis depends mainly on the hypnotic suggestibility of the subjects (Oakley and Halligan, 2013), whereas meditative traits can be developed with practice (Kiken et al., 2015). This difference affects experimental designs: while the effects of meditation can be assessed by comparing experienced and novice meditators, the effects of hypnosis are usually assessed by distinguishing between high and low hypnotizable individuals.

An important theoretical framework that highlights the differences between the cognitive mechanisms underlying these two states is the theory of Higher Order Thoughts (HOT; Rosenthal, 2005). In this theory, a mental state can be defined as conscious when a person is aware of living that mental state. In this sense, Dienes et al. (2020) proposed a differentiation between HOT cognitive control and COLD control, that is cognitive control in the absence of accurate metacognitive processes. This form of COLD control during hypnosis can be seen in the alteration of the sense of agency, defined as the experience of being the initiator of an intentional action. From this perspective, COLD hypnotic control is interpretable as a lack of awareness of intentions.

On the other end, in another set of studies in clinical settings and with inductions involving attention and imagery (Drigas et al., 2022), hypnosis appears to have an impact on metacognitive skills and wellbeing, thus opening up to the counterintuitive possibility that unconscious processes can act on metacognitive development.

Related to this, a crucial point of differentiation between hypnosis and meditation concerns the experience of dissociation, as the hypnosis literature agrees that a sense of involuntary action and dissociated volition is experienced during these states (Sadler and Woody, 2010). This appears to be very different from meditative states, where the emphasis is instead on increasing and integrating the meditator's sense of presence into one's experiences. Therefore, some researchers consider hypnosis a form of strategically self-induced deception, while meditation as a form of self-induced intuition (Dienes et al., 2016).

A final point of differentiation concerns the effect of these two states on the experience of being a self. While in hypnosis people report having some sort of hidden observer who was witnessing the suggested execution (e.g., motor response) from a third person perspective, in most meditative states practitioners are initially involved in an effort to observe the flow of their thoughts without being involved in it. As the practice progresses, meditators report a weakening of the first person perspective, to the point, in advanced meditative states, of a sense of dissolution of the boundaries of the self (Epstein, 1988).

## Direct experimental contrasts between hypnosis and meditation

Here, we review the studies that directly contrast hypnosis and meditation in experimental settings, arranging them into four different groups.

### Contrasting hypnosis and transcendental meditation

The first group includes four studies that compared hypnosis (self or hetero-induced) and TM. Walrath and Hamilton (1975) found no differences in heart rate, respiratory rate reduction, and skin resistance between two groups of experienced meditators who either performed a TM session or performed a self-hypnosis session.

Similar lack of physiological differences were found in Morse et al. (1977), in which participants were monitored during alertness, TM, hypnosis (with only relaxation or with analgesia) and relaxation while awake. Psychophysiological measurements included respiratory rate, heart rate, blood pressure, skin resistance, electroencephalography (EEG), and muscle activity. The results showed differences only with respect to alertness, while there were no significant differences between the states of relaxation, with the exception of muscle activity, which was deeper in meditation. Experientially, participants reported relaxation in hypnosis and meditation as being equally more effective than pure relaxation.

In a third study, Barmark and Gaunitz (1979) compared the effects of TM and audio-recorded hypnosis. As in the two previous studies, physiological data showed no significant differences between hypnosis and TM, particularly in heart rate and skin temperature. A slower respiratory rate was detected during TM. Participants reported that during hypnosis compared to alertness there was greater vividness in mental images and a heightened sense of concentration, along with less attention to environmental stimuli and respiratory sensations. Instead, during TM meditators reported that their bodies became lighter and warmer, and as if time went by faster. Benson et al. (1978) found that high hypnotizable subjects lowered anxiety and systolic blood pressure both in TM and self-hypnosis compared to lows.

### Contrasting hypnosis with attention meditation and open monitoring

A second group of studies compared hypnosis with attention and open monitoring meditation. In Nuys (1973), participants performed focused meditation exercises, attention assignments, and hypnotic susceptibility assessments. The results indicated that good concentration is a necessary condition for hypnotic susceptibility, but not sufficient, as some participants who did show good concentration were not suggestible. Spanos et al. (1978, 1980) replicated these results, reporting that hypnotizability is related to a low intrusion rate of distracting thoughts, and therefore to the absorption and vividness of mental images. Heide et al. (1980) showed that highly hypnotizable individuals presented most substantial decrements in anxiety after a 1-week meditation treatment compared to lows, while a brief training in meditation did not modify hypnotic responsivity.

Brown et al. (1983) conducted a study to investigate the phenomenological differences during self-hypnosis, daydreaming, and mindfulness meditation performed during retreats. While self-hypnosis involved more self-referential thinking, memory changes and intense emotions, daydreaming emphasized the presence of spontaneous mental images. Meditation initially involved a difficulty in managing distractions during practice, but with experience, a greater awareness of bodily processes was learned, facing changes in the perception of time and sense of self, with mental processes appearing to slow down and with a vivid awareness, which took on an impersonal quality.

More recently, Semmens-Wheeler and Dienes (2012) in the theoretical framework of HOT theory, underlined a methodological issue: the measurement of the subjective perception of intrusive thoughts is a self-monitoring (meta-awareness) activity, and it is therefore possible that highly hypnotizable individuals are simply not aware of the distracting intrusive thoughts. In this sense, the authors proposed the term “cold absorption” in the context of hypnosis, as opposed to “hot absorption” in experienced meditators. The authors compared the hypnotizability scores of experienced meditators against a database of 500 subjects, finding that the meditators were less suggestible than the average of all other subjects. They therefore hypothesized that meditation and hypnosis are opposite with regard to the role of meta-awareness. This hypothesis was verified in a survey by Lush et al. (2016), in which they investigated the subjective times of awareness of an intention to move, a judgment considered to be of a metacognitive type. They found that more easily hypnotized people are less capable of metacognitive judgment, and therefore attribute the initiation of the intention to move later than experienced meditators, whereas the practice of meditation leads to accurate judgments. Furthermore, a cross-sectional study (Grover et al., 2018) showed that hypnotizability and mindfulness facets where negatively correlated.

### Contrasting hypnosis and meditation in the perception of pain

A third group of studies compared hypnosis and meditation in the context of pain sensation, mostly for clinical treatments. In a recent review, De Benedittis (2021) underlines how both hypnosis and meditation attenuate pain, but with both similarities and differences in the multiple neurocognitive mechanisms involved. Both phenomena involve the frontal modulation of pain-related areas, but their role in hypnosis seems to depend on the type of suggestion given, while in meditation depends on the level of practice.

Swain and Trevena (2014) compared the effects of brief mindfulness and hypnosis sessions on resistance to pain caused by a hand placed in cold water at 0 C (Cold Pressor Task, CPT). Both interventions showed their efficacy compared to control in two different modalities: no difference was found on between DVD presentations and in person procedures. Participants, however, reported lower subjective pain scores after hypnosis compared to mindfulness.

Recently, Grover et al. (2021) replicated the previous study, finding no differences in CPT outcomes after a single recorded session of hypnosis or mindfulness meditation. Both conditions, however, modulated changes in self-reported pain perception, but while hypnosis induced a reduction in pain intensity and unpleasant elements of pain, mindfulness only correlated with a reduction in pain intensity.

Williams et al. (2022) evaluated the effectiveness of mindfulness meditation and hypnosis vs. an active control condition (educational training) in a randomized study of U.S. military veterans suffering from chronic pain and depression. The results showed no significant differences immediately after the treatments: however, in the follow-up evaluations at 3 and 6 months, the groups that practiced hypnosis and meditation showed a decrease in the intensity of pain and depression.

### Contrasting hypnosis and meditation in electroencephalographic studies

A fourth group of studies concerns hypnosis and meditation comparisons mainly with the use of EEG. Halsband et al. (2009) measured EEG activity during hypnosis and meditation of a single highly hypnotizable subject expert in Vajrayana practice, a form of Tibetan meditation that aims to achieve a state of enhanced cognition and emotions (Amihai and Kozhevnikov, 2014). They report significant differences between the two states in the alpha 1 and theta 2 frequency bands. High amplitudes in the alpha frequency bands were greater under hypnosis in the central and temporal positions, while the alpha frequency in meditation was more pronounced in the frontal positions than in the control. Greater activity in the theta band two was observed only under hypnosis in both hemispheres. While the authors admit that it is difficult to draw conclusions from a study with a single subject and with these variances, it is interesting to report that the two states do not show identical brain activations.

Another one-participant study compared the EEG correlates of one form of TM (Sidhi) with those caused by audio-recorded hypnosis in a man with moderate hypnotic responsiveness (Pekala and Creegan, 2020). The participant showed significant phenomenological differences between the two states, assessed by the Phenomenology of Consciousness Inventory, combined with electrophysiological correlates. A greater alpha and beta activity was found during TM than in hypnosis, with a greater beta in the left prefrontal cortex, and increased global delta activity during hypnosis.

Recently, intracranial EEG was used in three patients with no meditative or hypnotic experience (Bauer et al., 2022). The day after the surgery, patients listened to three different audios guiding to mind-wandering, mindfulness meditation, and an imaginative hypnotic state. The pre-recorded hypnotic procedure consisted of bringing attention to bodily sensations and then imagining visiting a pleasant place. The authors found non-specific and diffuse amplitude modulations in the three conditions. Connectivity analysis revealed common patterns in the three conditions, predominant in the low frequencies (delta, theta, and alpha). The connectivity patterns that were unique to the three conditions predominated in the gamma band, and one-third of the correlations in these models were negative.

## Conclusions and future directions

In summary, several theoretical models and some experiments identify points of overlap and points of difference between the hypnotic state and the meditative state. In particular, hypnosis appears to be a form of attention focalization supported by an external expert in suggestion methodologies, with prominent imaginative elements and with a dissociation of executive control. Meditative states induce a state of absorption and concentration, but these are typically self-induced, and are forced through numerous practice sessions, which over time can lead to a progressive integration into executive control and—in the long run—to a decrease in the differentiation between the self and the external world.

The large number of meditative practices and the many possible hypnotic inductions open to a combinatorial explosion of interesting experimental contrasts, many of which have not been yet performed. The experiments that directly investigated these similarities and differences are currently only preliminary, although they begin to show phenomenological differences involving metacognition, absorption and executive control, while psychophysiological and EEG studies are too few to draw any kind of meaningful conclusion.

To better understand these states of consciousness and their relationships with ordinary states, it is necessary to increase research efforts, both from the point of view of theoretical models and the collection of data that make a direct comparison between hypnosis and meditation.

## Author contributions

GP and ND contributed equally to the literature search, literature analysis, and writing of the manuscript. Both authors contributed to the article and approved the submitted version.

## Conflict of interest

The authors declare that the research was conducted in the absence of any commercial or financial relationships that could be construed as a potential conflict of interest.

## Publisher's note

All claims expressed in this article are solely those of the authors and do not necessarily represent those of their affiliated organizations, or those of the publisher, the editors and the reviewers. Any product that may be evaluated in this article, or claim that may be made by its manufacturer, is not guaranteed or endorsed by the publisher.
